# *Vibrio mimicus* Lineage Carrying Cholera Toxin and *Vibrio* Pathogenicity Island, United States and China

**DOI:** 10.3201/eid3008.240252

**Published:** 2024-08

**Authors:** Sergio Mascarenhas Morgado, Fernanda dos Santos Freitas, Erica Lourenço da Fonseca, Ana Carolina Paulo Vicente

**Affiliations:** Author affiliation: Instituto Oswaldo Cruz, Rio de Janeiro, Brazil

**Keywords:** vibrio, *Vibrio mimicus* lineages, bacteria, CTX, cholera toxin, virulence factor, outbreak, diarrhea, human infection, China, United States, food safety, enteric infections

## Abstract

*Vibrio mimicus* bacteria have caused sporadic cases and outbreaks of cholera-like diarrhea throughout the world, but the association of lineages with such events is unexplored. Genomic analyses revealed *V. mimicus* lineages carrying the virulence factors cholera toxin and toxin coregulated pilus, one of which has persisted for decades in China and the United States.

*Vibrio mimicus* bacteria are native to aquatic environments but have the potential to cause diseases in animals and humans, such as gastroenteritis and cholera-like diarrhea (*1*). In *Vibrio cholera* bacteria, the main toxigenic factor is cholera toxin (CTX), which is encoded by *ctx*A and *ctx*B genes and is part of the bacteriophage CTXФ. The acquisition of CTXФ phage by *V. cholerae* bacteria was associated with the toxin coregulated pilus (TCP), which is involved in intestinal colonization and aggregation. This virulence factor is encoded in an operon in the *Vibrio* pathogenicity island 1 (VPI-1), and *tcp*A is the main structural subunit of that pilus. In addition to TCP, VPI-1 also harbors the *acf*A-D operon, which also plays a role in colonization (*2*).

In 2004, the largest documented foodborne outbreak of *V. mimicus* occurred in Thailand, in which 306 persons experienced symptoms including diarrhea, abdominal pain, and vomiting (*3*), but the virulome associated with these strains was not verified. In 2010, in the United States, a cluster of severe diarrheal diseases was caused by *V. mimicus* strains carrying CTX (*4*). In 2019, *V. mimicus* bacteria caused a seafood-associated outbreak in Florida (USA), in which the patients experienced severe diarrhea, although the strains were CTX-negative (*5*). However, the virulome of most genomes analyzed (n = 33) was not explored, leaving a gap regarding the association of the strains or lineages with virulence factors. To fill this gap, we analyzed 44 *V. mimicus* genomes, 35 from GenBank and 9 environmental genomes from Brazil and Japan that we sequenced by using an Illumina Hiseq 2500, assembled by using SPAdes 3.15.2 (https://github.com/ablab/spades), and then analyzed by using Abricate (https://github.com/tseemann/abricate) and the Comprehensive Antibiotic Resistance Database (https://card.mcmaster.ca) and the Virulence Factor Database (http://www.mgc.ac.cn/VFs/main.htm).

The phylogenetic analysis based on the core genome revealed clusters; the 2 main clusters had genomes that had been circulating in China and the United States for decades (Figure). These 2 clusters are characterized by distinct virulomes; 1 co-harbored *ctx*A, *ctx*B, *ace*, *zot*, TCP, and *acf*A-D (mainly clinical genomes), whereas the other did not have any of those genes. The lineage carrying those virulence genes has persisted for >3 decades (1980–2009), infecting persons in China and the United States. The other lineage, which lacks these virulence factors, also was identified in China (2020) and the United States (1977 [human source]). Another interesting cluster of genomes is the one that covers Brazil (1998 [animal source]) and the United States (2016 [environmental source]). 

**Figure:**
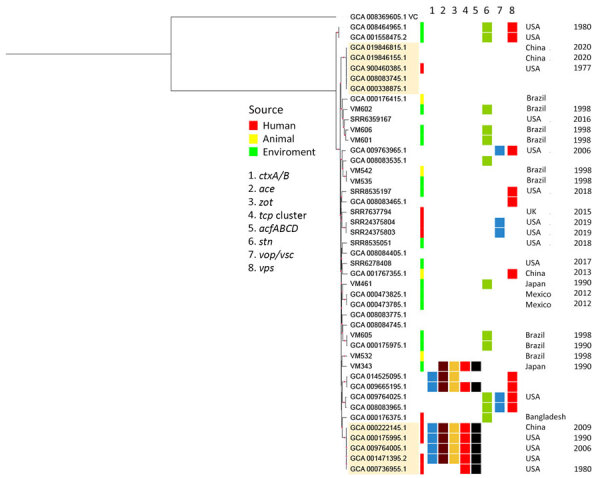
Maximum-likelihood phylogenetic tree of 44 *Vibrio mimicus* bacteria genomes from the United States and China. The best evolutionary model (general time reversible plus base frequencies plus ascertainment bias correction plus FreeRate model with 8 categories) was selected on the basis of the Bayesian information criterion. The beige highlighted clusters represent the main ones sharing genomes from China and the United States. Red circles on branches represent >70% bootstrap.

Our analysis indicates that *V. mimicus* lineages are disseminated and persist in distinct sources in space and time. In addition, another set of 3 related genomes (VM343, GCA_014525095.1, and GCA_009665195.1) also possessed CTX, TCP, or both, which suggests loss or partial acquisition of the pathogenicity islands of these elements. Of note, other genomes belonging to the same lineage (Figure) appear to have acquired the *ctx*A or *ctx*B genes from different sources; GCA_000175995.1, GCA_001471395.2, and GCA_009764005.1 (United States) possessed the *ctx*B2 genotype (El Tor [Australia]), whereas GCA_000222145.1 (China) had the *ctx*B1 genotype (classical [strain 569B]). Regarding the TCP cluster, analyses in blastn (https://blast.ncbi.nlm.nih.gov) revealed that all *tcp*A sequences, except for VM343, were identical and differed from the classical and El Tor genotypes. The *tcp*A allele carried by most genomes has also been characterized in nonpandemic clinical *V. cholerae* strains from the United States (*6*), whereas the *tcp*A allele of VM343 (Japan [environmental source]) is unique.

We identified several other virulence genes in the genomes (Appendix). We highlight the presence of the heat-stable enterotoxin gene (NAG-ST), identified throughout the phylogeny, and gene clusters related to exopolysaccharide production (*vps*) and the type III secretion system (T3SS) (*vop*, *vsc*, and *vcr*). We identified T3SS only in genomes that did not carry CTX, TCP, or both, including those from the 2019 outbreak in Florida (*5*), but 2 closely related genomes (1 of which was identified in the United States) co-carried the T3SS and NAG-ST genes. Because T3SS is a syringe-like protein secretion apparatus, the co-occurrence of this system with a diarrhea-associated toxin could increase the pathogenicity of these strains.

Our findings show that *V. mimicus* strains are spread throughout the world and that some of them carry a virulome comparable to that of *V. cholerae* bacteria. The virulome of environmental and clinical strains is apparently not heterogeneous (*5*), even with the analysis of the new environmental genomes. However, those findings may represent just the tip of the iceberg, given the bias regarding the locality of available genomes. Therefore, more genomic data must be generated to determine whether specialized clinical strains of *V. mimicus* exist, as they do for *V. cholerae* bacteria. Furthermore, the environmental and clinical genomes possessed a set of common virulence genes, suggesting that environmental strains have the potential to cause disease in humans. Because *V. mimicus* already possesses an intrinsic virulome, with the potential to cause disease, the acquisition of virulence determinants such as CTX, TCP, or both could specialize certain lineages, as revealed in our analysis by the clinical lineages that carry these determinants.

Appendix Additional information about *Vibrio mimicus* lineage carrying cholera toxin and *Vibrio* pathogenicity island, United States and China.
