# Supplementary material for: Vibrio mimicus Lineage Carrying Cholera Toxin and Vibrio Pathogenicity Island, United States and China
Source: Emerg Infect Dis. 2024 Aug;30(8):1729–32. doi: 10.3201/eid3008.240252 (PMC11286048; doi:10.3201/eid3008.240252)
Supplement: Appendix — Additional information about Vibrio mimicus lineage carrying cholera toxin and Vibrio pathogenicity island, United States and China. [file 24-0252-Techapp-s1.pdf]

Article DOI: https://doi.org/10.3201/eid3008.240252

EID cannot ensure accessibility for supplementary materials supplied by authors. Readers who have difficulty accessing supplementary content should contact the authors for assistance.

Vibrio mimicus Lineage Carrying Cholera Toxin and *Vibrio* Pathogenicity Island, United States and China

Appendix

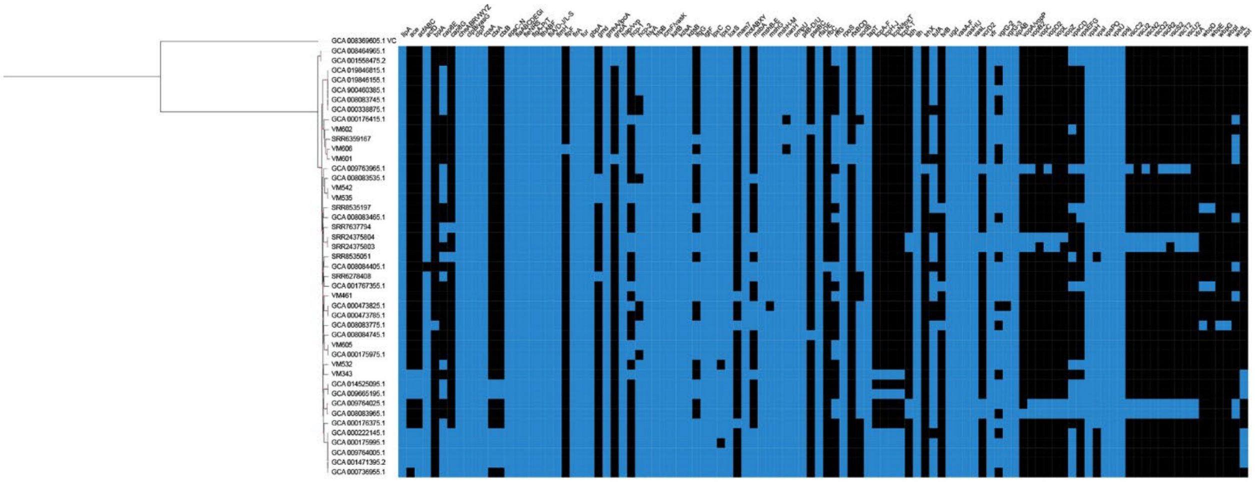

Figure S1. Heatmap of the virulence genes identified. Blue and black blocks indicate presence and absence, respectively

Appendix Figure. Heatmap of the virulence genes identified. Blue and black blocks indicate presence and absence, respectively.
